# Mutations in the Schmallenberg Virus Gc Glycoprotein Facilitate Cellular Protein Synthesis Shutoff and Restore Pathogenicity of NSs Deletion Mutants in Mice

**DOI:** 10.1128/JVI.00424-16

**Published:** 2016-05-12

**Authors:** Mariana Varela, Rute Maria Pinto, Marco Caporale, Ilaria M. Piras, Aislynn Taggart, Frauke Seehusen, Kerstin Hahn, Anna Janowicz, William Marciel de Souza, Wolfgang Baumgärtner, Xiaohong Shi, Massimo Palmarini

**Affiliations:** a MRC-University of Glasgow Centre for Virus Research, Glasgow, Scotland; b Istituto Zooprofilattico Sperimentale dell'Abruzzo e Molise G. Caporale, Teramo, Italy; c Department of Pathology and Center of Systems Neuroscience, University of Veterinary Medicine, Hannover, Germany; d Virology Research Center, Ribeirão Preto School of Medicine, University of São Paulo, Ribeirao Preto, Brazil

## Abstract

Serial passage of viruses in cell culture has been traditionally used to attenuate virulence and identify determinants of viral pathogenesis. In a previous study, we found that a strain of Schmallenberg virus (SBV) serially passaged in tissue culture (termed SBVp32) unexpectedly displayed increased pathogenicity in suckling mice compared to wild-type SBV. In this study, we mapped the determinants of SBVp32 virulence to the viral genome M segment. SBVp32 virulence is associated with the capacity of this virus to reach high titers in the brains of experimentally infected suckling mice. We also found that the Gc glycoprotein, encoded by the M segment of SBVp32, facilitates host cell protein shutoff *in vitro*. Interestingly, while the M segment of SBVp32 is a virulence factor, we found that the S segment of the same virus confers by itself an attenuated phenotype to wild-type SBV, as it has lost the ability to block the innate immune system of the host. Single mutations present in the Gc glycoprotein of SBVp32 are sufficient to compensate for both the attenuated phenotype of the SBVp32 S segment and the attenuated phenotype of NSs deletion mutants. Our data also indicate that the SBVp32 M segment does not act as an interferon (IFN) antagonist. Therefore, SBV mutants can retain pathogenicity even when they are unable to fully control the production of IFN by infected cells. Overall, this study suggests that the viral glycoprotein of orthobunyaviruses can compensate, at least in part, for the function of NSs. In addition, we also provide evidence that the induction of total cellular protein shutoff by SBV is determined by multiple viral proteins, while the ability to control the production of IFN maps to the NSs protein.

**IMPORTANCE** The identification of viral determinants of pathogenesis is key to the development of prophylactic and intervention measures. In this study, we found that the bunyavirus Gc glycoprotein is a virulence factor. Importantly, we show that mutations in the Gc glycoprotein can restore the pathogenicity of attenuated mutants resulting from deletions or mutations in the nonstructural protein NSs. Our findings highlight the fact that careful consideration should be taken when designing live attenuated vaccines based on deletions of nonstructural proteins since single mutations in the viral glycoproteins appear to revert attenuated mutants to virulent phenotypes.

## INTRODUCTION

The family Bunyaviridae is one of the largest families of RNA viruses, comprising pathogens of importance for both human and veterinary medicine. More than 170 viruses transmitted by arthropods form the Orthobunyavirus genus. Schmallenberg virus (SBV) is an orthobunyavirus of ruminants that emerged in central Europe in the summer of 2011 and spread very quickly throughout the rest of the continent (1). Although SBV genomes and antibodies have been detected in wild ruminants, camelids, and a dog, so far only infections of ruminants have been associated with the disease (2–4). SBV has been detected in various Culicoides species, and it is assumed that these insects provide the main route of transmission for this virus (5, 6).

Infection of adult animals with SBV results in unspecific and mild clinical signs, while infection during gestation can result in stillbirths, abortions, and malformations similarly to infections with related viruses of the Simbu serogroup, like Akabane virus (AKAV), Sathuperi virus (SATV), and Shamonda virus (SHAV) (7, 8). SBV was not detected in archived brain samples, and no evidence of antibodies toward this virus was found in sera collected before 2010 in ruminants (9, 10). Hence, it is believed that the virus emerged for the first time in Europe in 2011. However, there is little information on the viral genetic characteristics and ecological conditions driving SBV emergence.

Like other orthobunyaviruses, the SBV genome comprises three RNA segments of negative polarity, referred to as small (S), medium (M), and large (L). The S segment encodes the viral nucleocapsid and the nonstructural protein NSs in an overlapping reading frame. The M segment encodes the viral glycoproteins Gn and Gc, decorating the viral lipid bilayers, in addition to the NSm glycoprotein, a second nonstructural protein with poorly defined characteristics. The L segment encodes the viral RNA-dependent RNA polymerase (RdRp).

Using reverse genetics, we and others have previously shown that the SBV NSs protein is a determinant of pathogenesis (11–13). Deletion of the SBV NSs protein results in attenuation of pathogenicity in a suckling mouse model of infection. *In vitro*, NSs deletion (SBV-ΔNSs) leads to (i) impaired virus replication in interferon (IFN)-competent cells, (ii) an inability to inhibit IFN synthesis in infected cells, and (iii) an inability to induce cellular protein synthesis shutoff. The defects possessed by SBV-ΔNSs are the result, at least in part, of the incapacity of the mutated virus to induce the degradation/dephosphorylation of the cellular RNA polymerase II as a means of inhibiting cellular transcription and the IFN response of the host (12). Regulatory authorities in some European countries have granted provisional marketing authorization for an inactivated SBV vaccine (14). In addition, an NSs-NSm double-deletion SBV mutant has been proposed as a vaccine candidate, as it provides full protection in cattle upon wild-type virus challenge and does not induce viremia or clinical signs in cattle (15).

In an attempt to identify other viral determinants of pathogenicity, we serially passaged SBV in sheep IFN-incompetent cells (CPT-Tert), and the resulting virus, referred to as SBVp32, was unexpectedly more virulent in 3- and 7-day-old NIH-Swiss mice inoculated intracerebrally (11). Although SBVp32 displayed replication kinetics similar to those of wild-type SBV in CPT-Tert cells, it spread and induced pathological changes faster in the brains of suckling mice. We found that SBVp32 possesses nucleotide substitutions (most of which are nonsynonymous) in all the viral genes compared to wild-type SBV (11). The objective of this study was to identify the viral proteins associated with the increased virulence of SBVp32 in order to better understand orthobunyavirus pathogenesis. We found that the Gc glycoprotein of SBVp32 is a virulence determinant and facilitates cellular protein synthesis shutoff. In addition, SBVp32 Gc can compensate for the attenuated phenotype of NSs deletion mutants.

## MATERIALS AND METHODS

### Cell lines.

BSR-T7/5 cells (kindly provided by Karl Conzelmann) stably expressing the T7 polymerase were grown in Glasgow modified Eagle's medium supplemented with 10% fetal bovine serum (FBS), 10% tryptose phosphate broth, and G418 at a final concentration of 1 mg/ml. Sheep choroid plexus cells (CPT-Tert) (16) were grown in Iscove's modified Dulbecco's medium (IMDM) supplemented with 10% FBS. A549-ISRE-GFP cells expressing green fluorescent protein (GFP) under the control of an ISRE promoter (provided by R. Randall) (17) were cultured in Dulbecco's modified Eagle's medium (DMEM) supplemented with 10% FBS. All cell lines were cultured at 37°C in a 5% CO_2_ and 95% humidified atmosphere and were supplemented with penicillin and streptomycin.

### Isolation of primary fibroblasts.

Ovine fibroblasts were isolated from sheep ear skin. Briefly, the dermal layer was removed, cut into explants, and plated into dry tissue culture plates, dermis side up. The explants were incubated without medium for 1 h, and DMEM culture medium containing 10% FBS, penicillin and streptomycin, and 1% nystatin was then added. After sufficient outgrowth, the explants were discarded, and the remaining cells were cultured by using standard methods.

### Antibodies.

Antisera used in this study included a rabbit polyclonal antiserum against the SBV N protein (Proteintech) (11). Antibodies against γ-tubulin were obtained from Sigma, while antibodies against puromycin were obtained from Millipore (used at a 1:3,000 dilution). Peroxidase-labeled secondary antibodies against rabbit and mouse were purchased from GE Healthcare Life Sciences and Cell Signaling, respectively.

### Viruses.

All viruses used in this study were rescued by reverse genetics in BSR-T7/5 cells as previously described (11). Titers were determined by standard plaque assays performed in CPT-Tert cells. The reassortants derived in this study were named by including the segment corresponding to the SBVp32 strain. For example, SBV-S32 contains the S segment from SBVp32 and the L and M segments from wild-type SBV. SBVp32 refers to SBV passaged 32 times in cell culture. SBV-SML32 contains all the segments from SBVp32 but was derived by reverse genetics. A deletion mutant of the NSm protein was constructed by mutagenesis of the SBV M segment by deletion of amino acid residues 338 to 448 (X. Shi and R. M. Elliott, unpublished results).

### Labeling of nascent proteins with puromycin.

Monolayers of CPT-Tert cells at 30 to 40% confluence were infected with the indicated viruses at a multiplicity of infection (MOI) of 1. At 16 h postinfection, the medium was replaced with IMDM supplemented with 10 μg/ml puromycin dihydrochloride (Sigma) for 12 min. Cells were then washed with phosphate-buffered saline (PBS) and lysed by using 1× Laemmli buffer. SDS-PAGE and Western blotting were performed by using total cell lysates as previously described (18). For quantitative Western blotting, primary antibodies were detected by using peroxidase-labeled secondary antibodies with a ChemiDoc XRS^+^ scanner and quantified with Image Lab software (Bio-Rad). γ-Tubulin was used to normalize loading. All experiments were performed independently at least three times. For each virus, at least two independent virus preparations were used.

### Virus replication assays.

Virus replication kinetics were determined by infection of the indicated cell lines at an MOI of 0.001, unless stated otherwise, followed by titration of supernatants collected at different time points postinfection by limiting-dilution assays in BSR-T7/5 cells. Titers are expressed as the 50% tissue culture infectious dose (TCID_50_) (determined by the Reed-Muench method [31]). Each experiment was performed in triplicate and repeated three times using two different virus preparations.

### Immunofluorescence and confocal microscopy.

The indicated cell lines were infected with the indicated viruses and fixed with 5% formaldehyde, followed by immunofluorescence using a polyclonal antiserum toward the SBV N protein, as previously described (19, 20). Slides were analyzed by using a Leica GMIR2 confocal microscope.

### IFN bioassays.

Primary sheep fibroblasts were infected with the indicated reassortants (MOI of 1 or 0.5), and at 16 h postinfection, the supernatants were collected and clarified. UV-inactivated supernatants were used to stimulate A549-ISRE-GFP cells for 24 h when cells were fixed, and the number of GFP positive cells was determined by fluorescence-activated cell sorter (FACS) analysis. The amount of IFN present in each sample was estimated based on an IFN standard of a known concentration and expressed relative to SBV-ΔNSs.

### Quantification of viral mRNA.

Viral RNA was reverse transcribed by using an SBV-specific primer (5′-TTCGGCCCCAGGTGCAAATC-3′) with AccuScript HF reverse transcriptase according to the manufacturer's instructions, using 200 μg of total RNA. Two microliters of cDNA was used for quantitative reverse transcription-PCR (qPCR) (qRT-PCR) using Brilliant III Ultra Fast QPCR master mix as suggested by the manufacturer. The primers and probe used are as follows: SBV-S-FW (TCAGATTGTCATGCCCCTTGC), SBV-S-RW (TTCGGCCCCAGGTGCAAATC), and SBV-S-FAM (TTAAGGGATGCACCTGGGCCGATGGC). Reaction mixtures were cycled on a Stratagene Mx3005 qPCR system (Agilent Technologies), and data were analyzed with Mx3000P software.

### Histopathology and *in situ* hybridization.

Organ samples were fixed in formalin and embedded in paraffin using standard histological techniques. Slides were stained with hematoxylin and eosin (H&E).

*In situ* hybridization (ISH) to detect SBV mRNA was performed on all sections as described previously (21). Briefly, paraffin sections were dewaxed, hydrated, and washed in diethyl pyrocarbonate (DEPC)-treated water. After proteolytic digestion, postfixation, acetylation, and prehybridization, sections were hybridized overnight with a digoxigenin (DIG)-labeled probe (88 bp; 100 ng/ml) directed against the SBV nucleoprotein (21). Hybridized probes were detected by using an anti-DIG antibody conjugated with alkaline phosphatase and the substrates nitroblue tetrazolium chloride (NBT) and 5-bromo-4-chloro-3-indolyl phosphate (BCIP) (X-phosphate). SBV-positive and -negative animals as well as sections incubated with only hybridization buffer were included as controls.

### *In vivo* experiments.

Animal experiments were carried out at the Istituto Zooprofilattico Sperimentale dell'Abruzzo e del Molise G. Caporale (Teramo, Italy) in accordance with locally and nationally approved protocols regulating animal experimental use (protocol number 5383/2012). For survival studies, suckling NIH-Swiss mice (*n* = 10 to 15 per group) were inoculated intracerebrally with 400 PFU of the indicated reassortants/mutants and monitored daily for signs of disease for a period of 14 days. In order to test virus spread in the brain, 5-day-old NIH-Swiss mice (*n* = 3 per virus and time point) were inoculated intracranially with SBV, SBV-SML32, and SBV-M32 and euthanized at 8, 24, 48, and 72 h postinfection. Pathogenicities of SBV and SBVp32 were also compared in adult IFNAR^−/−^ mice, where groups of 5 mice were inoculated intraperitoneally (1,000 PFU) and weight was recorded over a 15-day period. For histology and ISH, IFNAR^−/−^ mice (*n* = 2 per virus) were inoculated with either SBV, SBVp32, or uninfected cell culture medium, and animals were euthanized at 3 days postinfection. Organs were then collected for histological examination and ISH.

### Statistical analysis.

Statistical analysis was performed by using GraphPad Prism. All graphs display data averages and standard deviations.

## RESULTS

### The M segment is a major determinant of SBVp32 virulence.

**FIG 1:**
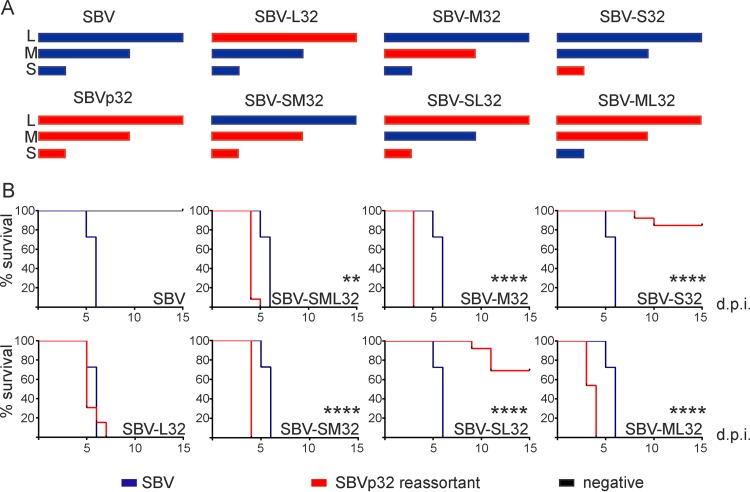
SBVp32 pathogenicity maps to the M segment. (A) Schematic representation of SBV reassortants generated by reverse genetics. (B) Survival plots of 11-day-old NIH-Swiss mice inoculated intracerebrally with 400 PFU of the indicated reassortants. Percent survival of mice inoculated with wild-type SBV is included in each panel to facilitate comparisons. Asterisks indicate significance levels (**, *P* ≤ 0.01; ****, *P* ≤ 0.0001 [as determined by a log rank test and a Mantel-Cox test]). d.p.i., days postinfection.

Our first goal was to identify the viral genomic segment(s) conferring higher virulence to SBVp32. To this end, we rescued SBV, SBVp32, and the reassortants between the two viruses mentioned below by reverse genetics as previously described (Fig. 1A) (11). Table 1 shows the mutations present in the viruses used in this study.

**TABLE 1:** Nucleotide differences between wild-type SBV and SBVp32^*a*^

Segment	SBV nt position		SBV nt	SBVp32 nt	Type of mutation		Location
	N	NSs			N	NSs	
S	66	41	C	T	Synonymous	Nonsynonymous	N/NSs
	124	99	G	A	Nonsynonymous	Synonymous	N/NSs
	167	142	A	G	Nonsynonymous	Nonsynonymous	N/NSs
	319	NA	A	G	Nonsynonymous	NA	N
M	1016		G	A	Nonsynonymous		NSm
	1239		T	G	Nonsynonymous		NSm
	1502		T	C	Nonsynonymous		Gc “hot spot”
	1894		A	G	Nonsynonymous		Gc
	2011		C	G	Synonymous		Gc
	2236		A	G	Nonsynonymous		Gc
	2411		C	T	Nonsynonymous		Gc
	2506		T	C	Nonsynonymous		Gc
	2575		G	A	Nonsynonymous		Gc
L	130		T	C	Nonsynonymous		Endonuclease region
	3044		T	C	Nonsynonymous		
	3858		A	G	Nonsynonymous		
	4078		C	T	Nonsynonymous		

a nt, nucleotide; NA, not applicable.

We inoculated 11-day-old NIH-Swiss mice intracerebrally (400 PFU) with all the reassortants, and survival was monitored for 15 days. All the reassortants carrying the M segment of SBVp32 were more pathogenic than wild-type SBV (*P* ≤ 0.01 for SBV-SML32 and *P* ≤ 0.0001 for SBV-M32, SBV-SM32, and SBM-SL32). These data suggest that the pathogenicity of SBVp32 maps to the M segment. A reassortant carrying the L segment of SBVp32 (SBV-L32) displayed the same pathogenicity as that of wild-type SBV; however, somewhat surprisingly, a reassortant carrying the S segment of SBVp32 (SBV-S32) was attenuated (Fig. 1B). It is interesting to note that the attenuation conferred by the S segment of SBVp32 could be compensated for by the M segment of SBVp32, since the SBV-SM32 mutant was more pathogenic than the wild type (*P* ≤ 0.0001).

**FIG 2:**
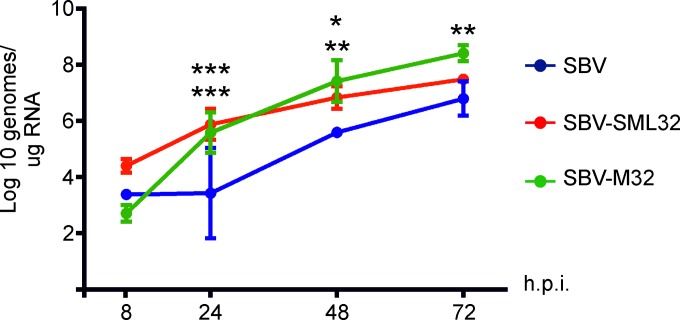
SBV carrying the M segment of SBVp32 reaches higher genome copy numbers in the brains of infected mice. Groups of 3 mice were infected intracranially, and brains were harvested at the indicated times postinfection. Viral genome equivalents were quantified by qRT-PCR. Data were analyzed by using 2-way analysis of variance (*, *P* ≤ 0.05; **, *P* ≤ 0.01; ***, *P* ≤ 0.001 [top asterisks indicate the significance level between SBV and SBV-SML32, while bottom asterisks indicate the significance level between SBV and SBV-M32]). h.p.i., hours postinfection.

We previously showed that SBVp32 spreads faster in the brains of infected mice (11). To understand the possible mechanisms behind the extra pathogenicity conferred by the M segment of SBVp32, we compared the amounts of virus found in brains of 6-day-old NIH-Swiss mice infected intracranially with wild-type SBV, SBV-SML32, and SBV-M32. For each virus, groups of 3 mice were killed at different times postinfection (8, 24, 48, and 72 h), and total RNA was extracted from brains, followed by qRT-PCR to quantify the number of SBV genome equivalents. We found that both SBV-SML32 and SBV-M32 reached higher numbers of viral genomes than those of wild-type SBV in the brains of infected mice (Fig. 2).

### SBV-S32 attenuation maps to a single nucleotide change within the S segment.

**FIG 3:**
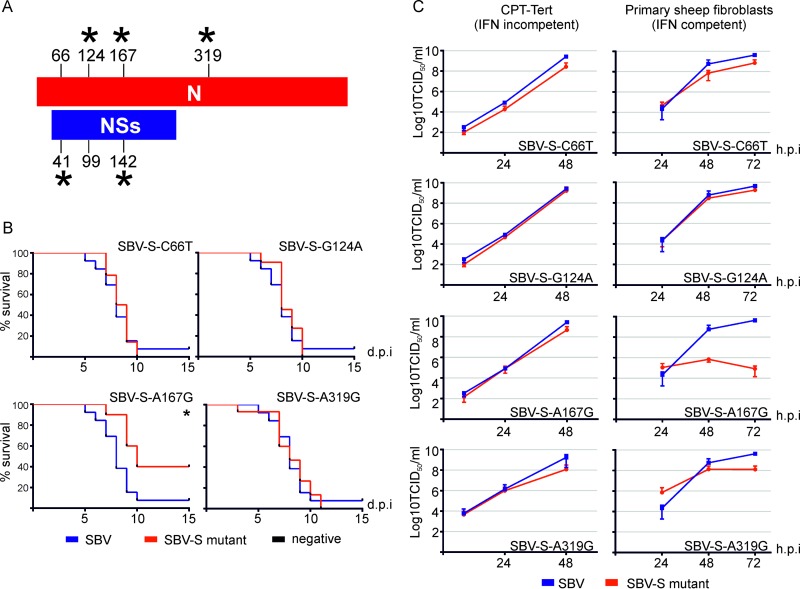
SBV-S32 attenuation maps to nucleotide 167/142 of the nucleocapsid/NSs gene. (A) Schematic representation displaying the positions of nucleotide changes of SBVp32 compared to wild-type SBV in the nucleocapsid and NSs open reading frames. Asterisks indicate nonsynonymous changes. (B) Survival plots of 7-day-old mice inoculated intracerebrally with 400 PFU of the indicated reassortants. Asterisks indicate significance levels (*, *P* ≤ 0.05 [as determined by a log rank test and a Mantel-Cox test]). (C) Replication kinetics of SBV S point mutants in CPT-Tert cells and primary sheep fibroblasts. The graph displays the averages of data from two and four independent experiments, respectively.

We first investigated the molecular determinants of the SBV-S32 attenuated phenotype. We derived 4 single mutant viruses (SBV-S-C66T, SBV-S-G124A, SBV-S-A167G, and SBV-S-A319G) where the nucleotide at position 66, 124, 167, or 319 of the SBV S segment was mutated to the corresponding nucleotide found in SBVp32, while the M and L segments were identical to those of wild-type SBV. These mutants were used to inoculate suckling mice. All the mutants killed 100% of mice similarly to wild-type SBV except for mutant SBV-S-A167G (carrying a G-to-A mutation corresponding to nucleotide 167 of the viral nucleocapsid gene and nucleotide 142 of the NSs gene), which was markedly attenuated (*P* ≤ 0.05) (Fig. 3A and B).

**FIG 4:**
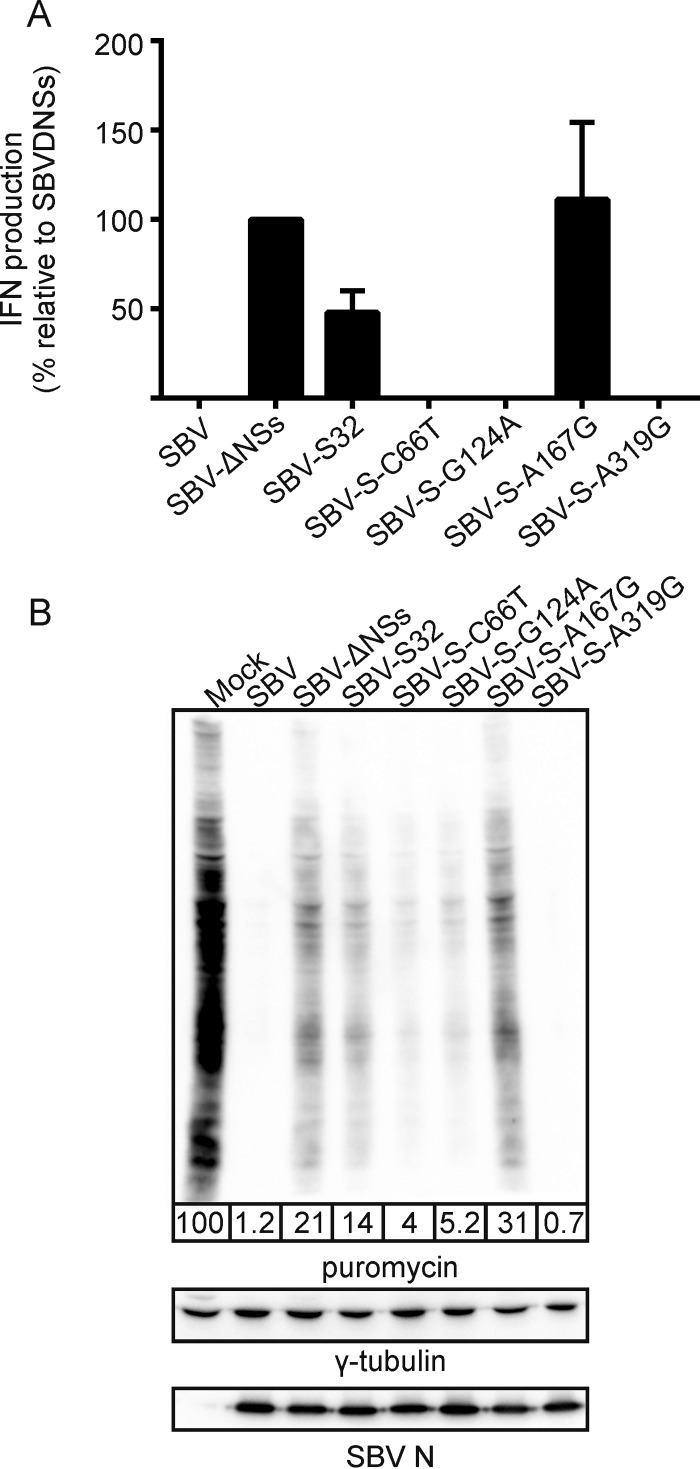
Mutation at nucleotide 167/142 of the nucleocapsid/NSs gene of the S segment of SBVp32 impairs the ability to induce total cellular protein shutoff and to control interferon production in infected cells. (A) Interferon bioassay. Primary sheep fibroblasts were infected with the indicated reassortants (MOI of 1), and the amount of IFN present in supernatants was estimated by comparing the ability to induce GFP expression in A549 cells stably expressing GFP under the control of an ISRE promoter to known quantities of universal IFN. Data are expressed relative to SBV-ΔNSs, which is known to induce IFN synthesis. (B) Western blots displaying puromycin-labeled proteins 16 h after infection with the indicated reassortants (MOI of 1). Infection with SBV-ΔNSs was used as control given its ability to shut down cellular protein synthesis. γ-Tubulin was used as a loading control, and SBV N was used to confirm infection. The numbers indicate the quantification of protein levels in each lane relative to that for mock infection, which was set at 100%.

While all the reassortants had replication kinetics similar to those of wild-type SBV in IFN-incompetent cells (CPT-Tert cells), replication of the SBV-S-A167G mutant was impaired in IFN-competent primary sheep fibroblasts (Fig. 3C). These data suggest that the attenuation of the S segment of SBVp32 due to mutation at position 167/142 (N/NSs) is related to the inability of this virus to inhibit the production of IFN. To confirm these data, we tested the ability of each mutant to induce IFN synthesis using an IFN bioassay. Primary sheep fibroblasts were infected with the different mutants, supernatants were collected at 16 h postinfection, and the amount of IFN present was estimated as described in Materials and Methods. As expected, no IFN was produced by cells infected with wild-type SBV, while cells infected with SBV-S32 and SBV-S-A167G induced the release of IFN into supernatants (Fig. 4A).

We have previously shown that the NSs protein of SBV counteracts the antiviral response of the host by inducing the shutoff of host cell protein synthesis, in line with other orthobunyaviruses (11–13, 22). To test if the attenuation of SBV-S32 is related to its inability to block cellular protein synthesis, we labeled nascent proteins with puromycin in cells infected with the mutants described above and compared them by Western blotting. Cells infected with SBV-ΔNSs were used as a control. SBV, SBV-S-C66T, SBV-S-G124A, and SBV-S-A319G induced host cell protein shutoff as expected. On the other hand, we found that SBV-S32 and SBV-S32-A167G were unable to induce total cellular protein shutoff, similarly to SBV lacking the NSs protein (SBV-ΔNSs) (Fig. 4B).

### The M segment of SBVp32 facilitates virus-induced shutoff of host cell protein synthesis.

**FIG 5:**
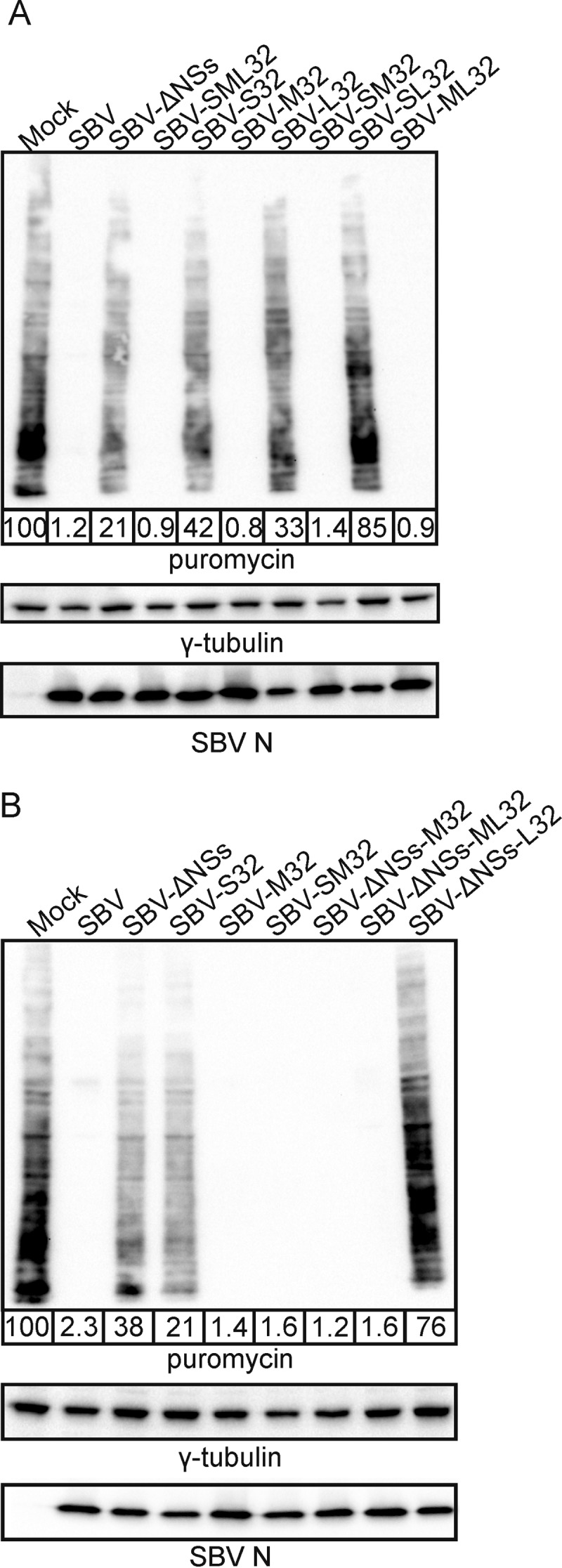
The M segment of SBVp32 rescues the inability of attenuated S segments to induce total cellular protein shutoff. Western blots display puromycin-labeled proteins 16 h after infection with the indicated reassortants (MOI of 1). γ-Tubulin was used as a loading control, and SBV N was used to confirm infection. The numbers indicate the quantification of protein levels in each lane relative to that for mock infection, which was set at 100%. (A) Reassortants carrying the S segment of SBVp32 in combination with wild-type or SBVp32 M and L segments. (B) Reassortants carrying a wild-type SBV S segment deleted of its NSs protein in combination with wild-type or SBVp32 M and L segments.

The results obtained so far indicated that SBVp32 is more virulent than wild-type SBV even though it harbors a defective S segment that is unable to counteract the IFN response of the host cell. However, the defect of the S segment can be compensated for, at least in the experimental mouse model used in our studies, by the M segment of SBVp32. Hence, we tested the ability of reassortants carrying the S segment of SBVp32 in combination with the L and M segments of SBVp32 to shut off protein synthesis by puromycin labeling of nascent proteins. These experiments were designed in order to understand whether the M segment of SBVp32 compensates for the relative inability of the SBVp32 S segment to block cellular protein synthesis. All the controls used in the experiment worked as expected: SBV induced host protein shutoff, while SBV-ΔNSs or SBV-S32 was unable to do so (Fig. 5A). On the other hand, we found that any reassortant carrying the M segment of SBVp32 was able to induce global host protein shutoff, even in the presence of the attenuated S32 segment. In addition, SBV-L32 was also unable to block host protein synthesis, suggesting a role for the polymerase in this process.

We then performed the same assays with reassortants deleted of the NSs protein in combination with the M or L segment of SBVp32 (SBV-SΔNSs-M32 and SBV-SΔNSs-L32). We found that the M segment of SBVp32 was also able to compensate for the defect in host protein shutoff displayed by an NSs deletion mutant (11) (Fig. 5B).

**FIG 6:**
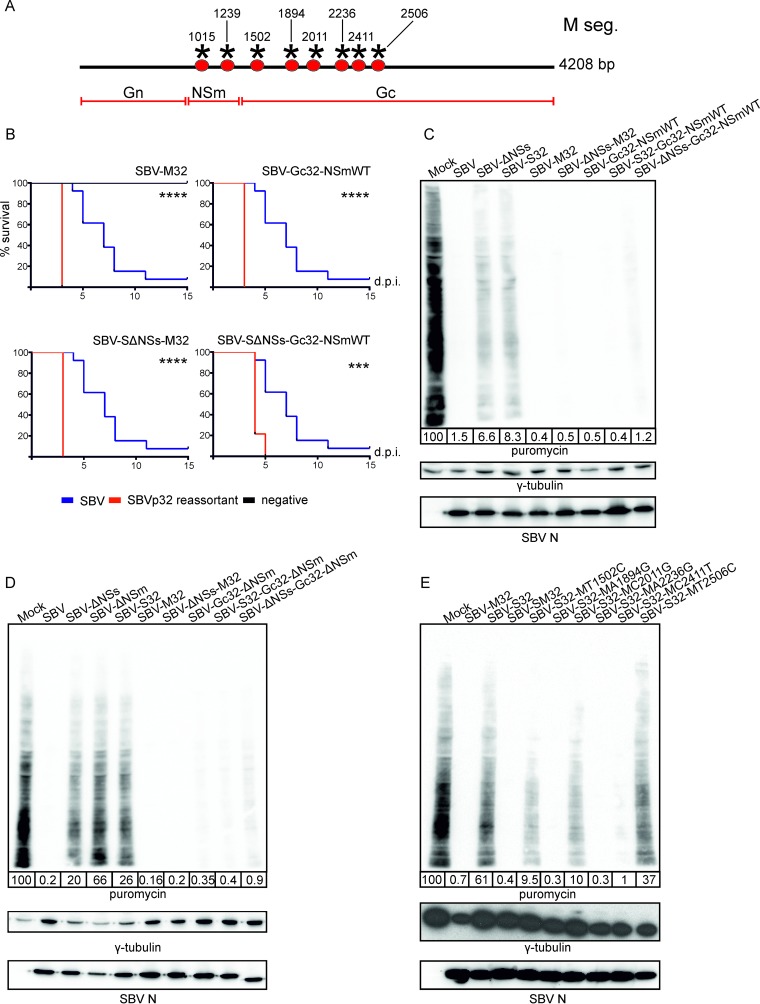
SBVp32 pathogenicity maps to the Gc glycoprotein. (A) Schematic representation of SBVp32 mutations within the M segment. (B) Survival plots of 8-day-old NIH-Swiss mice inoculated intracerebrally with 400 PFU of the indicated reassortants. Asterisks indicate significance levels (***, *P* ≤ 0.001; ****, *P* ≤ 0.0001 [as determined by a log rank test and a Mantel-Cox test). (C to E) Western blots displaying puromycin-labeled proteins 16 h after infection with the indicated reassortants (MOI of 1). γ-Tubulin was used as a loading control, and SBV N was used to confirm infection. The numbers indicate the quantification of protein levels in each lane relative to that for mock infection, which was set at 100%.

The mutations that accumulated in the M segment of SBVp32 during serial passage map to both the NSm and Gc glycoproteins (Fig. 6A and Table 1). To understand if the increased pathogenicity of SBVp32 maps to the NSm or the Gc glycoprotein, we mutated the NSm nucleotides within the SBV-M32 backbone into those present in wild-type SBV and tested the pathogenicity of the resulting reassortant in our mouse model of infection. We found that a virus carrying a wild-type NSm protein and an SBVp32 Gc protein in combination with L and S segments from wild-type SBV (SBV-Gc32-NSmWT) was more pathogenic than wild-type SBV in suckling mice (*P* ≤ 0.001) (Fig. 6B). In addition, the same reassortant in the context of an SBV S segment deleted of its NSs protein was still more pathogenic (SBV-SΔNSs-Gc32-NSmWT) than wild-type SBV (*P* ≤ 0.001).

Next, we checked the ability of these mutants to induce total cellular protein shutoff using puromycin labeling of nascent proteins. As expected, we found that all the reassortants carrying the Gc glycoprotein of SBVp32 induced total cellular shutoff even in combination with impaired S segments (S32 and SΔNSs) (Fig. 6C). In addition, complete deletion of the NSm protein did not affect the ability of the SBVp32 Gc glycoprotein to shut off total cellular protein production or restore the impaired cellular protein shutoff capability of NSs mutants (SBV-S32-Gc32-ΔNSm and SBV-ΔNSs-Gc32-ΔNSm) (Fig. 6D).

Next, we introduced the individual nonsynonymous nucleotide changes identified in the Gc gene of SBVp32 into SBV by site-directed mutagenesis and rescued each mutant within the context of a wild-type SBV L segment and the S segment of SBVp32. The resulting mutants were called SBV-S32-MT1502C, SBV-S32-MA1894G, SBV-S32-MC2011G, SBV-S32-MA2236G, SBV-S32-MC2411T, and SBV-S32-MT2506C. Unfortunately, SBV-S32-G2575A could not be successfully rescued despite several attempts. We then assessed the capacity of each mutant to shut off total cellular protein production. We found that mutations at positions 1894, 2236, and 2411 were individually capable of rescuing the defect of the S segment of SBVp32 (Fig. 6E).

### The M segment of SBVp32 is not an IFN antagonist.

**FIG 7:**
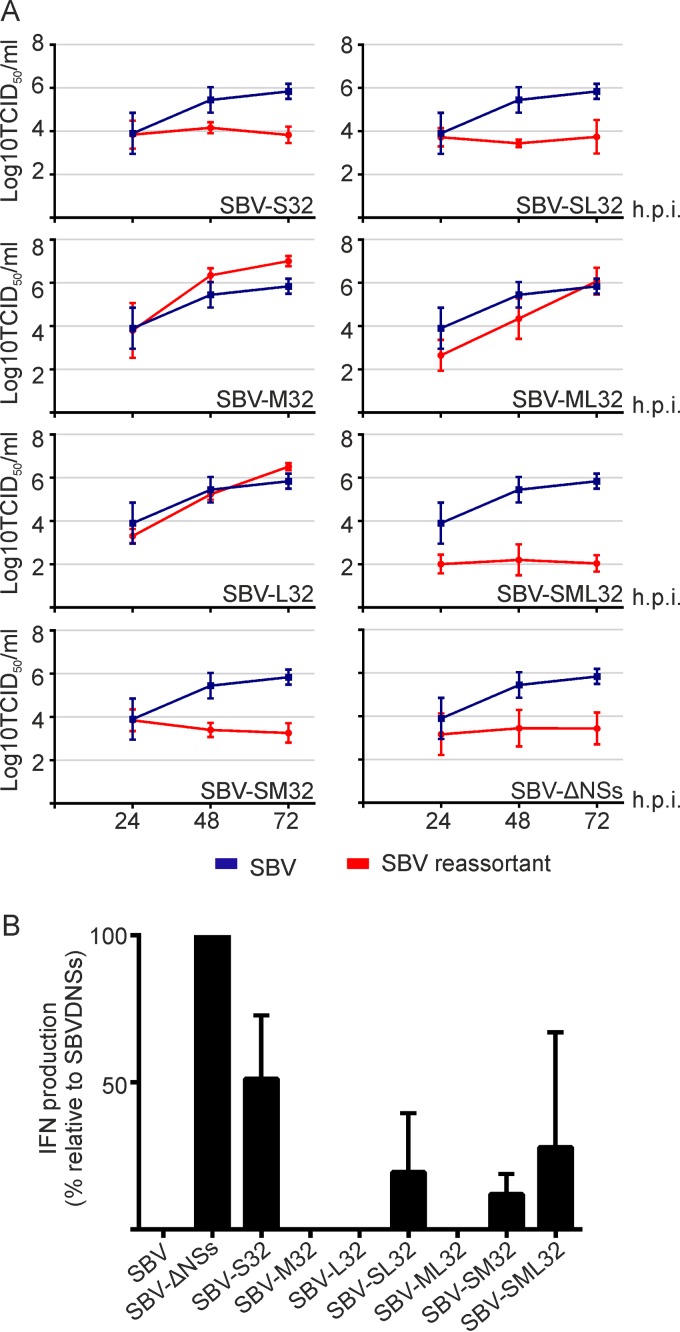
Replication kinetics of SBVp32 reassortants in IFN-competent cells. (A) Primary sheep fibroblasts were infected (MOI of 0.001) with the indicated reassortants, and samples were collected at the indicated times postinfection. The graph shows the averages of data from four independent experiments. (B) Interferon bioassay. Primary sheep fibroblasts were infected with the indicated reassortants (MOI of 0.5), and the amount of IFN present in supernatants was estimated as described in the legend of Fig. 4.

Next, we investigated whether the M segment of SBVp32 could compensate for the inability of the attenuated S32 segment to inhibit the production of IFN. To this end, we first monitored the replication kinetics of various SBVp32–wild-type SBV reassortants in sheep IFN-competent cells. We found that all reassortants carrying the S segment of SBVp32 (including those carrying M32) reached lower titers than those of wild-type SBV (Fig. 7A). These data suggest that the S segment of SBV is by itself the major virus IFN antagonist.

To support these results, we tested the ability of each reassortant to block the synthesis of IFN in infected cells as described above. We found that all the reassortants carrying the S segment of SBVp32 induced the release of IFN from infected cells, even in combination with the M segment of SBVp32, although at lower levels than those for an NSs deletion mutant (Fig. 7B). These data indicate that for SBV, pathogenicity does not totally depend on the capability of the virus to inhibit the production of IFN but rather depends on its ability to induce total cellular protein shutoff. In addition, the data also suggest that virus-induced global protein shutoff is not necessarily sufficient to block the production of IFN in the host.

### The glycoprotein of SBVp32 facilitates early events of virus infection.

**FIG 8:**
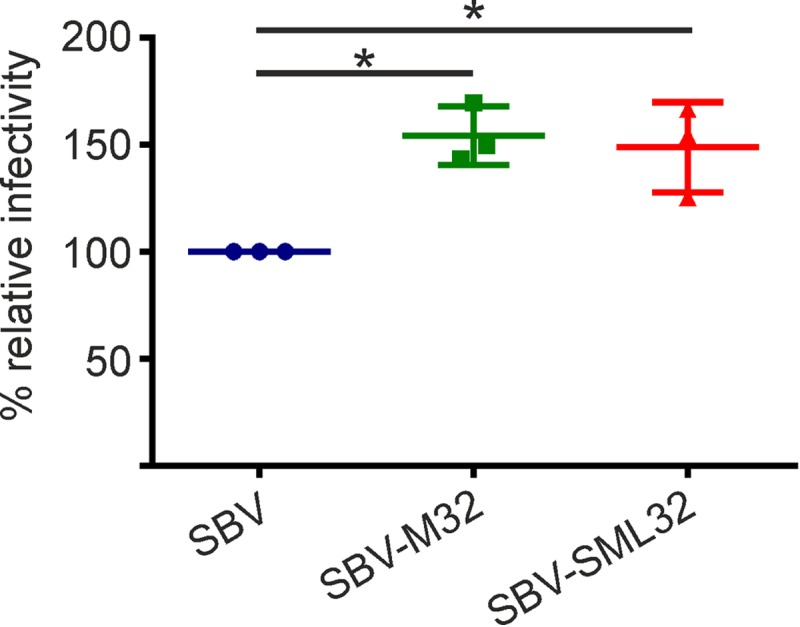
The SBVp32 glycoprotein is more infectious than the wild-type SBV glycoprotein. CPT-tert cells were infected with 2.5 × 10^5^ genome equivalents of the indicated viruses, and the number of SBV-positive cells was counted at 8 h postinfection after immune staining. Ten view fields were counted under each condition. The percentage of positive cells is presented relative to that of wild-type SBV infection. The experiment was repeated 3 times independently. Data were analyzed by using analysis of variance (*P* = 0.0076).

The majority of the amino acid substitutions between wild-type SBV and SBV-M32 map to the Gc glycoprotein. Therefore, we reasoned that during serial passage in tissue culture, the SBV glycoprotein evolved to reach a conformation to allow better entry. To test this hypothesis, we infected CPT-Tert cells with the same number of genome equivalents of SBV, SBV-M32, and SBV-SML32 (all segments from SBVp32), and the number of infected foci was quantified at 8 h postinfection after immune staining of the viral nucleocapsid protein. We found a higher number of SBV-positive foci in wells infected with SBVp32 and SBV-M32 than in those infected with wild-type SBV, indicating that the M segment of SBVp32 facilitates early events of SBV infection (Fig. 8).

### SBVp32 virulence is also related to increased virus replication in the absence of an intact IFN response.

**FIG 9:**
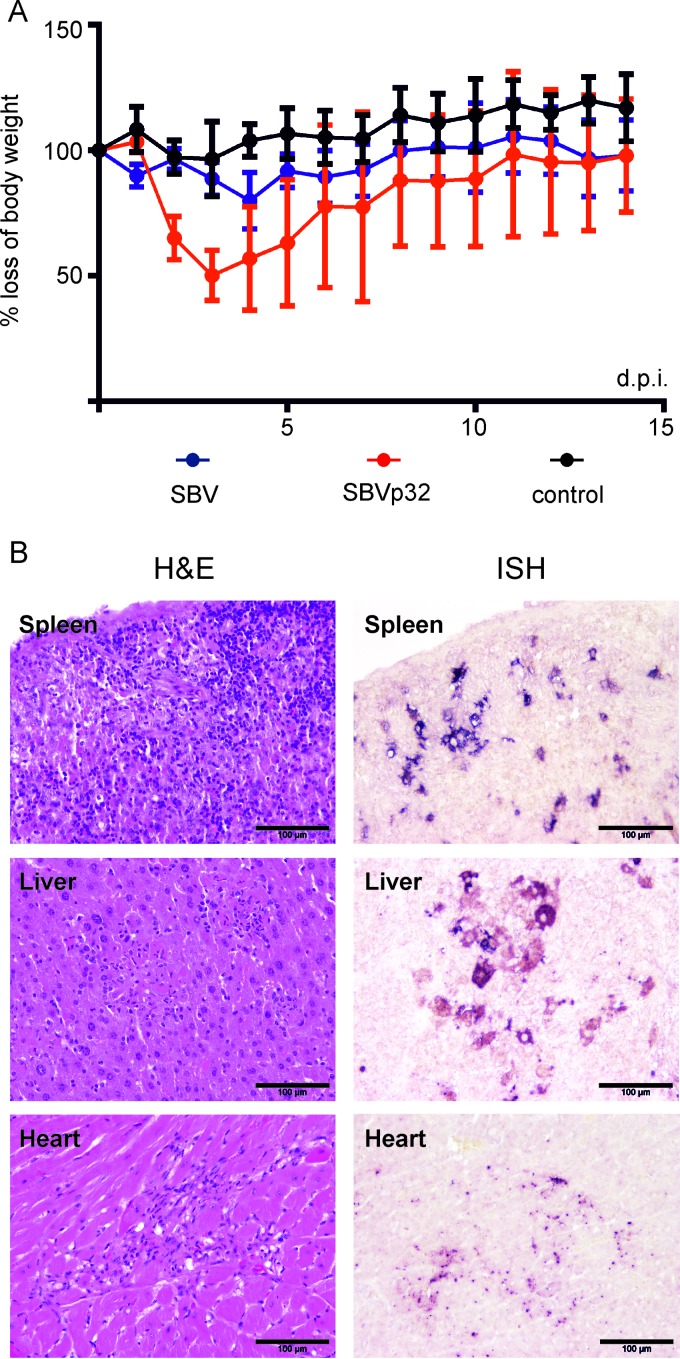
SBVp32 virulence is related to increased virus replication in IFNAR^−/−^ mice. (A) Groups of 5 mice were inoculated intraperitoneally (1,000 PFU), and weight was recorded over a 15-day period. The area under the curve was estimated for each mouse and then compared among groups (*P* < 0.05 [as determined by analysis of variance and a Tukey multiple-comparison test]). (B) Representative micrographs displaying the pathological changes observed in SBVp32-infected IFNAR^−/−^ mice (left) and the presence of viral RNA by ISH (right).

The data obtained so far indicate that SBVp32 virulence is determined by its M segment and in particular by its ability to shut off host protein synthesis and facilitate virus replication. The results obtained using NIH-Swiss mice suggest that SBVp32 virulence is therefore not linked to its ability to block the IFN response of the host. In order to support this conclusion, we compared the pathogenicities of SBV and SBVp32 in adult IFNAR^−/−^ mice. We inoculated three groups of IFNAR^−/−^ mice with SBV, SBVp32, or uninfected cell culture medium as a control (*n* = 5 per group). All the animals survived for the duration of the experiment, and both SBV- and SBVp32-infected animals developed signs of disease. However, these signs were more pronounced in SBVp32-infected animals. In addition, SBVp32-infected animals displayed statistically significant (*P* < 0.05) more pronounced weight loss than that in mock-infected controls (Fig. 9A).

Next, we analyzed virus distribution in a variety of organs by ISH in three groups of IFNAR^−/−^ mice (*n* = 2 per group) inoculated with either SBV, SBVp32, or uninfected cell culture medium. Mice were euthanized at 3 days postinfection, and organs were then collected for histological examination and ISH (Fig. 9B). The liver and spleen of SBV-infected animals were the only organs displaying histopathological changes, consisting of mild to moderate inflammation. SBV mRNA was detected by *in situ* hybridization in both macrophages and hepatocytes in the liver and in macrophages in the spleen. SBV mRNA was also detected in the mandibular and mesenteric lymph nodes. On the other hand, moderate to severe necrosuppurative hepatitis, suppurative splenitis, and lymphadenitis were found in samples derived from SBVp32-infected animals. These lesions were associated with the presence of moderate to large amounts of SBV mRNA in hepatocytes and macrophages. One animal showed nonsuppurative interstitial nephritis that was associated with the presence of mild to moderate amounts SBV mRNA. Degenerative and inflammatory lesions were identified in the heart, while mild pathomorphological changes were present in the small intestine and the spinal cord, but no SBV mRNA was detected in the latter. Table 2 displays the organs in which the presence of SBV mRNA was evaluated.

**TABLE 2:** Organs in which the presence of SBV mRNA was evaluated by *in situ* hybridization

Organ	Presence of mRNA^*a*^					
	SBV		SBVp32		Controls	
	Mouse 1	Mouse 2	Mouse 3	Mouse 4	Mouse 5	Mouse 6
Salivary gland	0	0	+	0	0	0
Mandibular lymph node	+	0	+++	+++++	0	ND
Pharynx	0	0	ND	0	0	0
Thyroid gland	0	ND	ND	0	ND	0
Lungs	0	0	0	0	0	0
Thymus	0	ND	ND	ND	ND	0
Heart	0	0	0	++	0	0
Liver	+++	0	++++++	++++++	0	0
Spleen	+++++	0	+++++	+++++	0	0
Kidney	0	0	+++	0	0	0
Testis	0	0	0	0	0	0
Cerebellum	0	0	0	0	0	0
Brain stem	0	0	0	0	0	0
Cerebrum	0	0	0	0	0	0
Stomach	0	0	0	0	0	0
Small intestine	0	0	+	++	0	0
Large intestine	0	0	0	++	0	0
Pancreas	0	0	0	0	0	0
Mesenteric lymph node	++	ND	+++++	ND	0	0
Spinal cord	0	0	0	0	0	0
Bone marrow	++++	0	+++++	+++++	0	0

a Mice 1 to 6 are individual animals. 0, no positive cells; +, single positive cells; ++, small number of positive cells; +++, small to moderate number of positive cells; ++++, moderate number of positive cells; +++++, moderate to large number of positive cells; ++++++, large number of positive cells; ND, not determined.

## DISCUSSION

In this study, we showed that a cell culture-adapted strain of SBV (SBVp32) has a more virulent phenotype than that of its wild-type parental strain due to amino acid substitutions in the Gc glycoprotein. Importantly, we show that SBVp32 virulence is increased by the capacity of Gc to facilitate host protein shutoff and early events of virus replication, at least in the experimental models employed in this study. Surprisingly, SBVp32 is more virulent than wild-type SBV but carries a defective S segment that is unable to inhibit the production of IFN and fails to induce total cellular protein shutoff in infected cells. This defect is, however, compensated for in SBVp32 by Gc. Altogether, our data indicate that the induction of total cellular protein shutoff in SBV is determined by multiple genes, while the ability to inhibit the production of IFN maps mainly to the NSs protein.

The S segment of SBV encodes the nucleocapsid and the nonstructural protein NSs in overlapping reading frames. We have previously shown that the SBV NSs protein is a virulence factor that inhibits the synthesis of IFN in infected cells and controls the innate immune system of the host by blocking cellular protein production (11, 12). Thus, it is not surprising that the S segment of SBVp32 lost the ability to repress the production of IFN given that it was serially passaged in CPT-Tert cells (sheep cells that do not to produce IFN upon viral infection). The NSs protein of SBVp32 also showed a decreased ability to induce total cellular protein shutoff. These observations suggest that IFN antagonism and the capacity to induce total cellular protein shutoff by the NSs protein are linked and require an arginine residue at position 49. Residue 49 is part of a nucleolar localization signal. Thus, the change from arginine to glycine in SBVp32 is likely associated with a change in the cellular localization of NSs leading to its loss of function and evidenced as an attenuated phenotype in suckling mice. However, pathogenicity is restored and even enhanced in those reassortants that also contain the Gc glycoprotein of SBVp32. The SBVp32 Gc protein acquired the ability to induce total cellular protein shutoff during serial passage but cannot compensate for the IFN antagonism of NSs. Altogether, these data suggest that the capacity to inhibit IFN production by SBV maps mainly to the NSs protein. On the other hand, SBV virulence is associated with the ability of the virus to induce total cellular protein shutoff rather than IFN antagonism. This is further supported by the fact that SBVp32 induces more severe clinical signs in adult IFNAR^−/−^ mice than those induced by wild-type SBV. These data also indicate that the ability to block IFN and induce cellular protein shutoff by the NSs protein comes with a “cost” for the virus that is easily disposed of once it is no longer required.

SBVp32 cannot fully inhibit the synthesis of IFN, and its replication is impaired in IFN-competent cells; however, it is still more pathogenic than wild-type SBV in suckling mice. A possible explanation for these contrasting observations is that in infections with viruses expressing the SBVp32 Gc protein, cellular shutoff precedes the induction of IFN, thus quickly overcoming the upregulation of IFN-stimulated genes (ISGs) and leading to pathogenicity. We need to take into consideration that we quantified the amount of IFN released into the supernatant of infected cells early in infection (16 h postinfection). It is possible that a different effect on IFN synthesis is seen at later time points during infection, and more studies will be required to address this point. In addition, the IFN bioassays were performed in primary cell cultures derived from adult animals. We have previously shown, as for other viral infections (23, 24), that there is an age-dependent resistance to SBV infection in suckling mice (11) that could be attributed to the development of anatomic barriers, the reticuloendothelial system, and the IFN and immune responses, and thus, the increased pathogenicity of SBVp32 could be due to many factors. It still remains to be determined if SBVp32 is also more pathogenic in ruminants and how this virus controls the innate immune system in its natural host.

Deletion of the NSs protein in wild-type SBV does not lead to a full recovery of protein expression in infected cells (Fig. 4B), indicating that other viral proteins are involved in inducing cellular protein shutoff. In addition, only eight serogroups encode NSs proteins among the viruses of the 15 orthobunyavirus serogroups for which genomic data are available, highlighting the fact that orthobunyaviruses use alternative mechanisms to cope with the innate immune system of the host (25). Here we observed that the L segment of SBV contributes to the induction of cellular protein shutoff given that a reassortant comprising the M and S segments of wild-type SBV and the L segment of SBVp32 (SBV-L32) was less capable of shutting down protein expression than wild-type SBV (Fig. 5A). Although we did not map the particular residues associated with this, we can speculate that a mutation at position 130 could be involved since it sits within the endonuclease motif of the viral polymerase. The endonuclease activity of the bunyavirus polymerase allows a “cap-snatching mechanism” by which the viral polymerase cleaves the 5′ cap of cellular mRNAs to use as primers for viral mRNA transcription, thereby reducing the cellular pools of mRNA for translation (26). We also found that the SBV NSm protein contributes to cellular protein shutoff (Fig. 4D). Interestingly, in another study, complete deletion of the NSm coding region resulted in a virus that, within the context of wild-type M and L segments (SBV-ΔNSm), was unable to induce cellular protein shutoff but had no effect on the ability to control the production of IFN in infected cells (R. M. Elliott and X. Shi, personal communication). This is in agreement with results of studies carried out on Bunyamwera virus (BUNV), where deletions of internal domains of NSm resulted in a similar phenotype, although a complete NSm deletion mutant could not be rescued by reverse genetics (27), suggesting that NSm might play different roles in different orthobunyaviruses. Although BUNV NSm has been involved in playing a role in assembly and morphogenesis, its role in controlling the innate immunity of the host requires further investigation.

The molecular mechanism of SBV Gc-induced protein shutoff remains to be determined. However, given that the Gc glycoproteins of other orthobunyaviruses, including SBV, transit from the endoplasmic reticulum (ER) to the Golgi apparatus (28) (not shown), it can be expected that they trigger the ER stress response. Several viruses use this strategy to favor the translation of viral proteins (29). Viral infection can quickly lead to excess protein and the accumulation of misfolded products in the ER, which ultimately results in the phosphorylation of eukaryotic initiation factor 2 (eIF2). eIF2 phosphorylation results in cap-dependent protein synthesis shutoff, relieving the burden of the accumulation of proteins in the ER and evidenced as a reduction in total cellular protein expression (30).

Reassortants carrying the SBVp32 M segments reached higher virus titers in the brains of infected mice and were more infectious *in vitro*. We hypothesize that during serial virus passage and without pressure from neutralizing antibodies, the Gc protein accumulated mutations that allow more efficient entry and, thus, faster spread. However, it remains to be determined if this virus could be viable or if it could be more pathogenic in its natural hosts given the possibility that it could be better neutralized. It is possible that faster entry or higher-level replication conferred by the SBVp32 Gc glycoprotein allows earlier expression of other viral proteins (such as the viral polymerase and/or NSm), leading to faster induction of cellular protein shutoff than in the wild-type virus. Unfortunately, we have not been able to express the viral glycoproteins to high levels using a transient-transfection system to test the role of Gc in protein shutoff independently of other viral components. These data could be an indication that expression of Gc leads to protein synthesis shutoff resulting in the repression of its own plasmid.

We showed that single point mutations in Gc (positions 1894, 2236, and 2411) rescue the inability of the S segment of SBVp32 to induce total cellular protein shutoff. There are currently <10 SBV M segment sequences derived from field isolates; thus, our understanding of the true genetic diversity of this virus and the consensus sequence circulating in affected countries is very limited. Interestingly, SBVp32 mutation at position 2236 exists in the field, indicating that it can lead to viable virus. Given that we found a consistent positive correlation between the ability of our reassortants to induce protein shutoff and the ability of the reassortants to be more pathogenic in our mouse model of infection, we can conclude that a single amino acid change in the Gc glycoprotein can potentially rescue attenuated phenotypes based on mutations of the S segment. Importantly, we also showed that the defect in inducing host protein shutoff by an NSs-NSm double-deletion mutant can be rescued by amino acid changes in the Gc glycoprotein and that at least one of the changes exists in viruses circulating in the field. Therefore, live attenuated vaccines based on deletions of NSs and NSm require further careful consideration.
